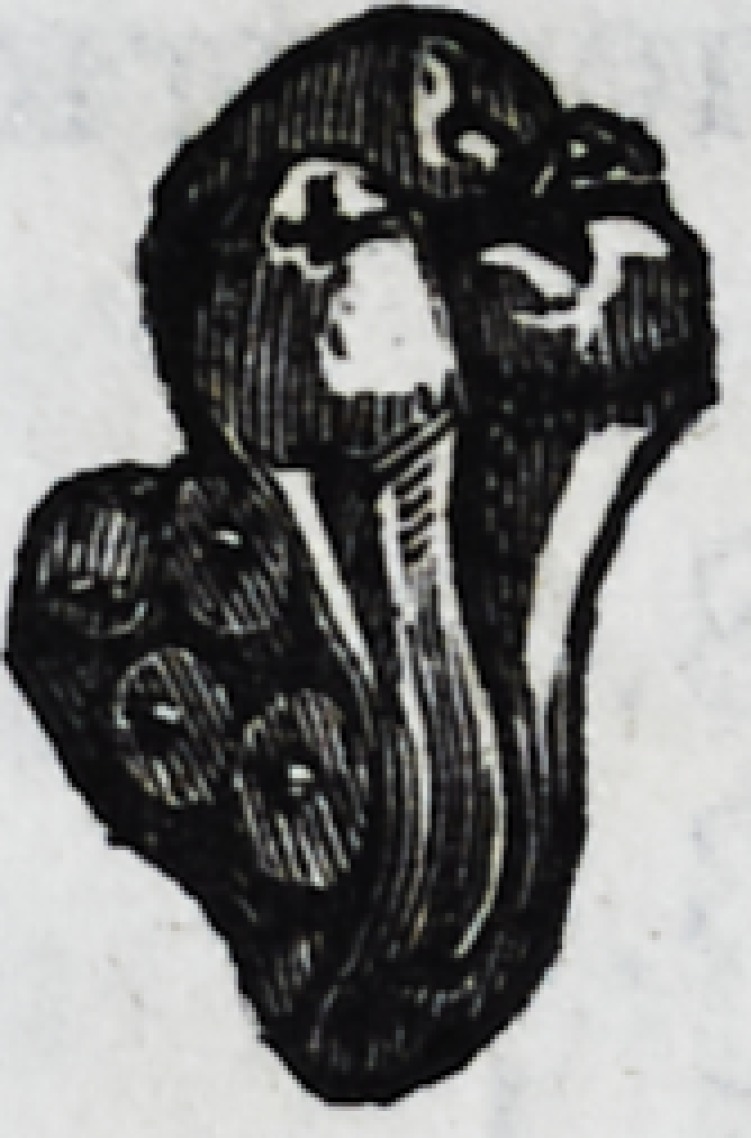# Extraordinary Successive Development of Teeth

**Published:** 1858-01

**Authors:** E. H. Andrews

**Affiliations:** Charlotte, Mecklinburg Co., N. C.


### ARTICLE III.

### *Extraordinary Successive Development of Teeth.*

IN October last, Mr. R. A. Plummer, of this county, called to have some teeth extracted. I saw that the right superior incisor was wanting. I asked him why he did not have it replaced? He said that could not be, that teeth continued to grow out, and already ten or twelve had been extracted from that space. He then showed me one (as he thought) just making its appearance, and said, that in six or eight months it would be out; that in a few months thereafter it would become loose, and that he would have to extract it.

About the middle of July he called again to have another tooth extracted; which I removed, and then prevailed upon him to let me take out what I thought would turn out to be the fang of the superior incisor. But, to my astonishment, it proved to be a mass of eight teeth in a cluster. These teeth were making their appearance wrong end foremost; as the discolored part was that which had made its appearance through the gums.

Mr. P. has no recollection of any injury having been done to his teeth when a child.

CHARLOTTE, *Mecklinburg Co., N. C.*

*August* 1, 1857.

E. H. ANDREWS.

I neglected to say that Mr. P. is a respectable man, whose word may be relied upon; and also, at this time another tooth is making its appearance in place of the cluster I took away.

## Figures and Tables

**Figure f1:**